# Current State of Pharmacogenomic Implementation Into Care for Persons With Cystic Fibrosis

**DOI:** 10.1002/ppul.71229

**Published:** 2025-08-08

**Authors:** Emma M. Tillman, Cameron McKinzie, Dave Young, Charissa Kam

**Affiliations:** ^1^ Division of Clinical Pharmacology Indiana University School of Medicine Indianapolis Indiana USA; ^2^ Department of Pharmacy University of North Carolina Medical Center Chapel Hill North Carolina USA; ^3^ Department of Pharmacotherapy at the University of Utah College of Pharmacy Salt Lake City Utah USA

**Keywords:** adverse drug reactions, cystic fibrosis, drug interactions, pharmacogenomics

## Abstract

Cystic fibrosis (CF) was once a fatal disease of childhood, but with advances in combination CFTR modulator therapies, life expectancy for persons with CF (PwCF) has increased. Despite remarkable improvements in life expectancy, CF is a chronic multiple organ system disease and comorbidities characterized by recurrent respiratory infections, pancreatic insufficiency, diabetes, liver disease, depression, anxiety, and bone disease resulting in exposure to many drugs. The Clinical Pharmacogenetics (PGx) Implementation Consortium (CPIC) publishes evidence‐based guidelines for use of PGx to guide dosing for drug−gene interactions. This study aimed to assess the current use of PGx testing in CF care at CF Foundation‐accredited care centers and affiliate programs (CFF‐ACCAP) across the United States. A 14‐item survey was distributed electronically to CF Foundation‐accredited care centers and affiliate programs in the United States using the CF Foundation email exchange. Overall, 74 responses were received from a potential of the 287 CFF‐ACCAP. Since each individual CFF‐ACCAP may have had multiple team members who could have received and responded to the survey, it is possible that these responses include multiple respondents from a single center. Only eight (4%) respondents affirmed they were obtaining PGx testing beyond cystic fibrosis transmembrane conductance regulator (CFTR) and 66 (89%) respondents answered that they were not currently doing PGx for drug−gene pairs beyond CFTR. Based on this landscape survey, PGx is not commonly implemented in US CFF‐ACCAP, but providers are open to using PGx to improve care in PwCF. Several barriers limit the implementation of PGx in CFF‐ACCAP, which calls for guidance on how to effectively integrate PGx into CF clinical care.

## 1 Introduction

Cystic fibrosis (CF) was once known as a fatal disease of childhood, but now with advances in cystic fibrosis transmembrane conductance regulator (CFTR) modulator therapies, anti‐infective, and airway clearance regimens, children born between 2020 and 2024 have a median predicted life expectancy of 65 years, nearly doubling over the life expectancy over the past two decades [1, 2, 3, 4, 5]. Despite these remarkable improvements in life expectancy, CF is a chronic multiple organ system disease characterized by recurrent respiratory infections, pancreatic insufficiency, diabetes, liver disease, metabolic bone disease, and comorbidities such as depression and anxiety [6, 7]. As a result of this disease sequelae, people with CF (PwCF) are often exposed to many medications in their lifetime with patients age 12−17 years receiving a mean of 17 unique prescriptions [8]. Additionally, with extended life expectancy, PwCF are expected to have increasing needs for medications associated with an aging population, such as cardiovascular disease medications.

Before initiating CFTR modulator therapy, all PwCF have CFTR genotype testing completed [9]. In addition to CFTR modulators, many medications prescribed to PwCF have Clinical Pharmacogenetics (PGx) Implementation Consortium (CPIC) evidence‐based guidelines for use of PGx to guide dosing with gene−drug interactions [7]. PGx has demonstrated the ability to improve medication outcomes in both clinical trials [10, 11, 12, 13, 14] and real‐world settings [15, 16, 17]. In a study at Indiana University Health, the authors found that of the 576 PwCF seen at the CF center in a 16‐year period, 504 (87.5%) received at least one drug that could have been dosed according to CPIC guidelines, if PGx results had been available [18]. In this study, the most commonly utilized drugs in PwCF with CPIC guidelines included: opioids, nonsteroidal anti‐inflammatory, selective‐serotonin reuptake inhibitors, tricyclic antidepressants, proton pump inhibitors, and serotonin 5‐HT_3_ receptor antagonist [18]. The potential utility for pharmacogenomics in CF care has been described by investigators at both adult and pediatric CF centers [19, 20, 21]. Despite standard of care CFTR genotype testing [9] and high utilization of drugs with CPIC guidelines that could be used to tailor therapy to improve efficacy and minimize toxicity, preemptive PGx testing in not standard of care in PwCF. The aim of this study was to assess the current use of PGx testing in CF care at CF Foundation‐accredited care centers and affiliate programs (CFF‐ACCAP) across the United States.

## 2 Methods

Following exempt institutional review board approval, an electronic survey was designed and adapted from previous PGx surveys [22]. CFF‐ACCAP were identified via the US (Cystic Fibrosis Foundation [CFF]) database. The survey was distributed via the CFF‐ACCAP center director, advanced practice provider (APP), and pharmacist‐pharmacy technician e‐mail exchanges to optimize the survey response rate. Although the survey was sent to many discipline listservs and there was a potential to receive multiple responses from the same center, the investigators were interested in responses from multiple disciplines and how these responses might correlate or differ. The survey was sent on November 15, 2024, and respondents were given 4 weeks to complete the survey (survey closed on December 13, 2024). A reminder was sent out on Week 2 during that period via e‐mail. Completion and submission of the survey served as the individual's consent to participate in the study. The survey included questions about the demographics of the CF center, as well as knowledge, awareness, and implementation of PGx. The time to complete the survey was estimated to be 10 min.

Participants were asked whether pharmacogenomic (PGx) testing beyond CFTR genotyping was used in their practice, and if so, whether it was performed proactively—before initiating medications potentially affected by PGx variants—or reactively, in response to reduced efficacy or adverse effects from such medications. Participants were asked to select all barriers that limit their ability to use PGx testing in clinical practice includingcost, logistical challenges, interpreting and implementing results, and lack of guidance specific to care of PwCF.

Following this initial section, participants were asked about implementation, defined as: PGx testing that is completed and yields results for clinical use (e.g., entered into the health record) which may include infrastructure in place to support test ordering, test interpretation, applying results to drug prescribing, and/or returning results to patients. Participants were asked about the population that they have implemented PGx testing in; PGx implementation infrastructure available at their center; where genotyping was performed; which genes were tested; what references they utilize to assist in interpreting PGx results; and presence of PGx computer decision support (CDS); or precision medicine clinical services to assist with PGx interpretation and guidance.

The full survey can be found in the supplementary data file. All responses were sorted and categorized by the role of the participant submitting the survey. Data was analyzed using descriptive statistics.

## 3 Results

The electronic survey was sent, and 74 recipients completed the survey in an average time of 17.5 min. Of these, 72 were self‐described as primary CF centers and 2 were affiliate CF programs, with 33 (45%) pediatric centers, 22 (30%) adult centers, and 19 (26%) both pediatric and adult. Respondents included 17 (23%) CF center directors, 24 (32%) physicians, 7 (9%) APPs, 25 (34%) pharmacists, and one respondent (1%) selected other. Forty‐two (57%) respondents affirmed that their CF center has a pharmacist involved in patient care, seven (9%) confirmed that no pharmacist is involved in patient care at their center, and 25 (33%) respondents did not answer this question. Full survey demographics are shown in Table 1.

**Table 1:** Survey demographics.

	*N* = 74 (%)
Type of center	
Primary center	72 (97)
Affiliate program	2 (3)
Center population	
Pediatric	33 (45)
Adult	22 (30)
Pediatric and adult	19 (26)
Discipline	
Pharmacist	25 (34)
Physician	24 (32)
CF center director	17 (23)
Advanced practice practicioner (NP/PA)	7 (9)
Other	1 (1)

### 3.1 CF Centers Currently Implementing PGx

Respondents were surveyed about all aspects of pharmacogenomics. Full survey data and responses are shown in Table 2, and Table 3 includes the survey responses delineated by provider type. When asked if PGx testing for other drug−gene pairs is obtained at their center, only eight (11%) respondents affirmed they were obtaining PGx testing beyond CFTR, and 66 (89%) respondents answered no. Of these eight respondents, one (1%) reported proactive testing, three (4%) reported reactive testing, and four (5%) reported a mix of both proactive and reactive PGx testing. When asked about barriers to PGx testing, 17 responses were provided (multiple reasons could be selected) including: five responses noted lack of guidance specific to care of PwCF; four (5%) citing cost of testing; four (5%) reporting challenges with interpreting and implementing results; three (4%) cited logistical challenges with obtaining PGx tesing; and one (1%) respondent marked other. When asked specifically about the stage of PGx implementation, 13 (18%) respondents affirmed that at least one drug−gene pair has been implemented; two (3%) selected that they were in the planning stages for implementation; and 58 (78%) respondents selected that PGx were not performed as part of clinical care. The most common PGx genes tested were CYP3A4 (*n* = 10), CYP3A5 (*n* = 9), CYP2D6, CYP2C9, CYP2C19 (*n* = 7), and UGT1A1 (*n* = 4). Fourty‐five (61%) answered that they did not know which PGx genes were implemented, and “Other” was chosen by 11 (15%) respondents. Only six (8%) respondents confirmed that they had a formal PGx consult service; 20 (27%) respondents have a PGx clinical pharmacist; four (5%) responded that they had electronic medical record (EMR) based CDS, and 23 (31%) cited other expertise or decision making tools that assist with PGx implementation.

**Table 2:** Pharmacogenomics survey with results.

Question	Responses	Overall (*N* = 74)
1. Are you currently familiar with pharmacogenomics and its use in clinical practice?	a. No, I don′t know what pharmacogenomics is.	5 (7)
	b. Yes, I know what pharmacogenomics is, but am not familiar with its clinical application.	14 (19)
	c. Yes, I know what pharmacogenomics is and its use in clinical practice, but do not currently use it in my own practice.	42 (57)
	d. Yes, I know what pharmacogenomics is and its use in clinical practice, and have experience with using it in my own practice	13 (17)
2. Other than CFTR genotyping, is your CF center obtaining PGx testing for any drug−gene pairs?	a. Yes (branch to #3)	8 (11)
	b. No (branch to #5)	66 (89)
3. In your clinical practice, do you obtain PGx testing proactively or reactively?	a. Proactive	1 (12)
	b. Reactive	3 (38)
	c. Mixture of a and b	4 (50)
4. What current barriers, if any, do you perceive that limit your ability to use PGx testing in your clinical practice? (Select ALL that apply)	a. Cost of testing	4 (50)
	b. Logistical challenges	3 (38)
	c. Challenges with interpreting/implementing results	4 (50)
	d. Lack of CF‐specific guidance	5 (63)
	e. Other (please specify)	1 (12)
5. Based on this definition, would you consider proactive or reactive PGx testing valuable in your clinical practice?	a. Proactive (proceed to #6)	5 (8)
	b. Reactive (proceed to #6)	16 (24)
	c. Both a and b (proceed to #6)	44 (67)
	d. Would not be valuable (proceed to #7)	0 (0)
	e. Other (please specify)	1 (2)
6. What barriers, if any, do you perceive that would limit your ability to use PGx testing in your clinical practice?	a. Cost of testing	58 (88)
	b. Logistical challenges	56 (85)
	c. Challenges with interpreting/implementing results	48 (73)
	d. Lack of CF‐specific guidance	3 (45)
	e. Other (please specify)	0 (0)
7. Why do you think PGx testing would not be valuable and/or beneficial to your current practice?	a. Cost of testing	0 (0)
	b. Logistical challenges	0 (0)
	c. Challenges with interpreting/implementing results	0 (0)
	d. Lack of CF‐specific guidance	0 (0)
	e. Other (please specify)	0 (0)
8. Is PGx‐guided implementation for at least one drug−gene pair implemented or planned at your institution?	a. Currently implemented	13 (18)
	b. Planning stage	2 (3)
	c. Not implemented (research only)	58 (78)
9. Which patient populations receive (or will receive) PGx‐guided implementation?	a. Adult patients	13 (18)
	b. Pediatric patients	11(17)
	c. Both	26 (35)
10. How long has PGx testing infrastructure been available at your institution?	a. ≤ 6 months	2 (3)
	b. > 6 months–1 year	2 (3)
	c. > 1–5 years	6 (8)
	d. > 5 years	3 (4)
	e. I do not know	17 (23)
	f. Not currently implemented	39 (53)
11. Where is genotyping performed at your institution? (Select ALL that apply)	a. In‐house	13 (18)
	b. Other healthcare institution lab	3 (4)
	c. Commercial lab	23 (31)
	d. Research lab	5 (7)
	e. I do not know	33 (45)
	f. Other (please specify)	3 (4)
12. Which genes are tested and used to guide therapy? (Select ALL that apply)	a. CYP2D6	7 (9)
	b. CYP2C9	7 (9)
	c. CYP2C19	7 (9)
	d. CYP3A5	9 (12)
	e. CYP3A4	10 (14)
	f. UGT1A1	4 (9)
	g. I do not know	45 (61)
	h. Other (please specify)	11 (15)
13. What resources/references do you use to interpret PGx results? (Select ALL that apply)	a. CPIC guidelines	19 (26)
	b. PharmGKB	10 (14)
	c. PubMed	21 (28)
	d. Tertiary references	10 (14)
	e. Commercial lab guidance	17 (23)
	f. Other (please specify)	13 (18)
14. What decision support/expertise is available to interpret PGx results? (Select ALL that apply)	a. PGx consult service	6 (8)
	b. PGx clinical pharmacist	20 (27)
	c. EMR‐based support	4 (5)
	d. Other (please specify)	23 (31)

**Table 3:** Pharmacogenomics survey with results, stratified by provider type.

Question	Responses	Physician (*N* = 41, %)	Pharmacist (*N* = 26, %)	APP (*N* = 7, %)
1. Are you currently familiar with pharmacogenomics and its use in clinical practice?	a. No, I don′t know what pharmacogenomics is.	4 (9.5)	0 (0)	1 (14)
	b. Yes, I know what pharmacogenomics is, but am not familiar with its clinical application.	8 (19.5)	3 (12)	3 (43)
	c. Yes, I know what pharmacogenomics is and its use in clinical practice, but do not currently use it in my own practice.	20 (49)	19 (73)	3 (43)
	d. Yes, I know what pharmacogenomics is and its use in clinical practice, and have experience with using it in my own practice.	9 (22)	4 (15)	0 (0)
2. Other than CFTR genotyping, is your CF center obtaining PGx testing for any drug−gene pairs?	a. Yes (branch to #3)	5 (12)	3 (12)	0 (0)
	b. No (branch to #5)	36 (88)	23 (88)	6 (86)
3. In your clinical practice, do you obtain PGx testing proactively or reactively?	a. Proactive	0 (0)	1 (4)	N/A
	b. Reactive	3 (7)	0 (0)	
	c. Mixture of a and b	2 (5)	2 (8)	
4. What current barriers, if any, do you perceive that limit your ability to use PGx testing in your clinical practice? (Select ALL that apply)	a. Cost of testing	1 (2)	3 (12)	N/A
	b. Logistical challenges	3 (7)	0 (0)	
	c. Challenges with interpreting/implementing results	4 (10)	0 (0)	
	d. Lack of CF‐specific guidance	5 (12)	0 (0)	
	e. Other (please specify)	0 (0)	0 (0)	
5. Based on this definition, would you consider proactive or reactive PGx testing valuable in your clinical practice?	a. Proactive (proceed to #6)	2 (5)	2 (8)	1 (14)
	b. Reactive (proceed to #6)	4 (10)	10 (38)	2 (29)
	c. Both a and b (proceed to #6)	30 (73)	11 (42)	3 (43)
	d. Would not be valuable (proceed to #7)	0 (0)	0 (0)	0 (0)
	e. Other (please specify)	0 (0)	0 (0)	1 (14)
6. What barriers, if any, do you perceive that would limit your ability to use PGx testing in your clinical practice?	a. Cost of testing	30 (73)	21 (81)	7 (100)
	b. Logistical challenges	31 (76)	19 (73)	6 (86)
	c. Challenges with interpreting/implementing results	25 (61)	17 (65)	6 (86)
	d. Lack of CF‐specific guidance	25 (61)	17 (65)	6 (86)
	e. Other (please specify)	3 (7)	2 (8)	0 (0)
7. Why do you think PGx testing would not be valuable and/or beneficial to your current practice?	a. Cost of testing	0 (0)	0 (0)	0 (0)
	b. Logistical challenges	0 (0)	0 (0)	0 (0)
	c. Challenges with interpreting/implementing results	0 (0)	0 (0)	0 (0)
	d. Lack of CF‐specific guidance	0 (0)	0 (0)	0 (0)
	e. Other (please specify)	0 (0)	0 (0)	0 (0)
8. Is PGx‐guided implementation for at least one drug−gene pair implemented or planned at your institution?	a. Currently implemented	7 (17)	6 (23)	0 (0)
	b. Planning stage	1 (2)	0 (0)	1 (14)
	c. Not implemented (research only)	32 (78)	20 (77)	6 (86)
9. Which patient populations receive (or will receive) PGx‐guided implementation?	a. Adult patients	7 (17)	5 (19)	1 (14)
	b. Pediatric patients	8 (20)	2 (8)	2 (29)
	c. Both	16 (39)	10 (38)	5 (71)
10. How long has PGx testing infrastructure been available at your institution?	a. ≤ 6 months	1 (2)	1 (4)	0 (0)
	b. > 6 months–1 year	2 (5)	0 (0)	0 (0)
	c. > 1–5 years	4 (10)	2 (8)	0 (0)
	d. > 5 years	0 (0)	3 (12)	0 (0)
	e. I do not know	10 (24)	5 (19)	2 (29)
	f. Not currently implemented	22 (54)	14 (54)	3 (43)
11. Where is genotyping performed at your institution? (Select ALL that apply)	a. In‐house	7 (27)	6 (23)	0 (0)
	b. Other healthcare institution lab	2 (5)	2 (8)	0 (0)
	c. Commercial lab	19 (46)	2 (8)	2 (29)
	d. Research lab	3 (73)	1 (4)	1 (14)
	e. I do not know	15 (37)	14 (54)	4 (57)
	f. Other (please specify)	1 (2)	0 (0)	0 (0)
12. Which genes are tested and used to guide therapy? (Select ALL that apply)	a. CYP2D6	2 (5)	5 (19)	0 (0)
	b. CYP2C9	2 (5)	5 (19)	0 (0)
	c. CYP2C19	2 (5)	5 (19)	0 (0)
	d. CYP3A4	3 (7)	5 (19)	1 (14)
	e. CYP3A5	3 (7)	5 (19)	0 (0)
	f. UGT1A1	2 (5)	2 (8)	0 (0)
	I do not know	27 (66)	13 (50)	5 (71)
	h. Other (please specify)	0 (0)	3 (12)	0 (0)
13. What resources/references do you use to interpret PGx results? (Select ALL that apply)	a. CPIC guidelines	8 (31)	10 (38)	1 (14)
	b. PharmGKB	3 (7)	5 (19)	1 (14)
	c. PubMed	12 (29)	8 (31)	1 (14)
	d. Tertiary references	5 (12)	4 (15)	1 (14)
	e. Commercial lab guidance	11 (27)	5 (19)	1 (14)
	f. Other (please specify)	2 (5)	1 (4)	0 (0)
14. What decision	a. PGx consult service	1 (2)	4 (15)	0 (0)
	b. PGx clinical pharmacist	11 (27)	7 (27)	1 (14)
Support/expertise is available to interpret PGx results? (Select ALL that apply)	c. EMR‐based support	0 (0)	4 (15)	0 (0)
	d. Other (please specify)	4 (10)	1 (4)	0 (0)

### 3.2 CF Centers That Have Not Implemented PGx

**Figure 1:**
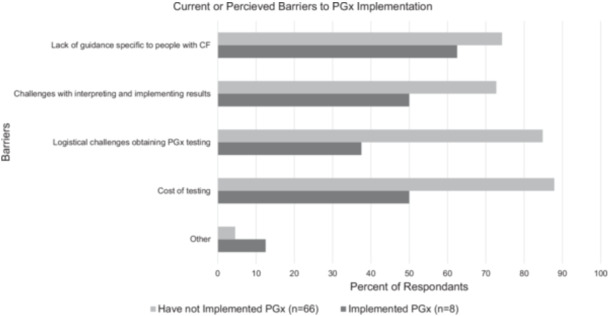
Current or perceived barriers to PGx implementation.

When asked if they would consider PGx implementation at their CF center, 65 (88%) confirmed they would consider implementing, with five (7%) considering proactive testing, 16 (22%) considering reactive testing, and 44 (59%) both proactive and reactive. No respondents selected that PGx would not be valuable, but one (1%) respondent selected “Other” and eight (11%) respondents did not answer, but we could assume that these were the eight that have already implemented PGx in their CF practice. Respondents were asked to select all current or perceived barriers for PGx implementation and these are shown compared to the PGx implementers' responses in Figure 1.

### 3.3 Institutional Infrastructure for PGx Implementation

We asked respondents questions about the institutional infrastructure that was available to support PGx implementation. Three (4%) respondents affirmed that infrastructure for PGx implemention has been available for greater than 5 years; six (8%) respondents selected > 1–5 years of infrastructure; two (3%) selected > 6 months –1 year; two (3%) selected < 6 months, 17 (23%) did not know, and 39 (53%) had no infrastructure. When asked where PGx testing was performed, the majority of 33 (45%) respondents did not know where PGx testing was performed.

## 4 Discussion

This study is the first comprehensive survey that has addressed the current state of PGx implementation in CFF‐ACCAP in the United States. Our data demonstrate three key findings: (1) PGx is not commonly implemented in US CFF‐ACCAP; (2) Providers are open to using PGx to improve care in PwCF; (3) Several barriers limit the implementation of PGx in US CFF‐ACCAP.

Despite the current standard of care of CFTR genotyping for every PwCF to confirm eligibility for CFTR modulator therapy, few CFF‐ACCAP are implementing PGx testing beyond CFTR genotyping. In the past few years, several CFF‐ACCAP have reported their use of PGx to improve care for PwCF. The University of Utah Health Care System implemented PGx testing for CYP2C19, CYP2C8, CYP2C9, CYP2D6, CYP3A4, and CYP3A5 in 52 PwCF [20]. An order for at least one actionable PGx medication was observed in 75% of the cases enabling 4.2 treatment modifications per 10 PwCF [20]. The authors noted that CYP2D6 and CYP2C19 were the most common genes related to drug therapy problems [20]. In our survey, CYP3A4 was the most commonly reported gene tested, although CPIC only has evidence for guiding tacrolimus dosing in conjunction with CYP3A5 [23]. While CYP3A4 has less clinical PGx‐guided therapy information, it is likely the most familiar on the list of metabolizing enzymes and could be an indicator of biased or inaccurate data reporting.

Indiana University has reported both the potential benefit of PGx implementation and experience implementing PGx in their CF population [18, 24]. To assess the potential impact of PGx testing in PwCF, investigators at Indiana University evaluated the medical records of 576 PwCF treated at their institution over a 16‐year period [18]. Eighty‐seven percent (504) of PwCF received at least one drug that could have been dosed according to CPIC guidelines, if PGx results would have been available [18]. Furthermore, in a retrospective study of 184 PwCF treated with selective serotonin reuptake inhibitors (SSRIs), 45% experienced SSRI treatment failure [24]. Only 44 PwCF had PGx results, and of these, only nine had actionable genotypes corresponding with the SSRI prescribed. Of these persons with PGx results and actionable genotypes, they had an SSRI failure rate of 78% (7 of 9) which was significantly higher compared to 45% (83 of 184) of the total cohort [25].

Although few CFF‐ACCAP are implementing PGx, it was very encouraging that respondents were overwhelmingly interested in using PGx to improve care. It is estimated that ~20%−30% of ADRs could be avoided if PGx data were known at the time of prescribing [25]. Furthermore, adverse drug reactions (ADRs) are three times more likely to occur in children when compared to adults, and up to 95% of ADRs go unreported [26, 27, 28]. Therefore, early PGx testing of children with CF could not only improve care of these children, but the results would be readily available as they age and are exposed to more medications with PGx recommendations. Although this reduction of ADRs has not been validated by clinical trials in PwCF, knowing genotype at the time of prescribing has the potential to avoid ADRs.

Our survey identified several barriers limiting the implementation of PGx in CFF‐ACCAP, including a lack of CF‐specific recommendations on how to utilize test results, infrastructure for PGx testing, and the cost of testing. Notably, the CFF‐ACCAP that have published research and/or implementation of PGx are associated with large academic medical centers with strong PGx implementation programs [20, 24, 29]. As the use of genetic data, including PGx, becomes increasingly more common in practice [29, 30], CFF‐ACCAP needs to adapt to this change in practice and learn how to best implement and utilize PGx to guide pharmacotherapy.

Understanding the coverage of PGx testing continues to be a major challenge for implementation. Although PGx coverage is variable ranging from 36% to 48% for single gene testing and upwards of 74% for panel testing [31], reimbursement rates continue to improve as more data for use emerges. As of July 2024, 29 states have introduced or passed legislation requiring health plans to provide coverage for biomarker testing that will aid in diagnosis, prevention, or treatment of disease [32]. Currently, 15 states require state‐regulated health plans to cover biomarker testing; five states require some plans to cover testing; and nine states have introduced, but not yet passed legislation [32]. While these bills were initially introduced to justify coverage for cancer biomarker testing, the general verbiage used in this legislation opens the door for justification for any pharmacogenomic test that could be used to prevent an ADR. This would make PGx testing more accessible and affordable, thereby expanding access to precision medicine to prevent, diagnose, and treat disease.

This study is not without limitations. Although we received responses from 26% (74 of 287) of CFF‐ACCAP, our results may be biased in favor of centers that have implemented PGx, as a center that was unfamiliar with PGx may have been more likely to not respond to the survey. Selection bias and sample size are limitations that we must acknowledge. Although the survey was sent to multiple listservs and there was potential for more than one response per center, based on center size, demographics, and answers to questions, there were no duplicate results. If there were more than one response from a center, the answers provided were not the same. Furthermore, the purpose of the survey was to describe current use and understanding of PGx among CF providers and care team members. Thus, members even within the same care center may have different experiences and comfort levels with PGx, and this was captured in this study. In addition, CFF‐ACCAP that have completed extensive PGx implementation were likely highly motivated to complete the survey to the end.

## 5 Conclusions

It is clear from this survey that the CF community is highly interested in using PGx to improve the care of their PwCF, yet they seek guidance and recommendations specific to the CF population. As research and implementation of PGx in PwCF continue to expand, a growing body of evidence now supports its use and provides recommendations for integrating PGx into CF care. Our group has proposed working with the CF Foundation to develop a position statement for the use of PGx in PwCF to provide guidance to clinicians. Additionally, we would like to survey PwCF to assess their interest and/or awareness of the benefits of PGx testing.

## Author Contributions

**Emma M. Tillman:** conceptualization, investigation, writing – original draft, methodology, data curation, project administration, writing – review and editing. **Cameron McKinzie:** conceptualization, investigation, methodology, writing – review and editing, data curation, formal analysis. **Dave Young:** conceptualization, investigation, writing – review and editing, methodology, formal analysis, data curation. **Charissa Kam:** formal analysis, investigation, conceptualization, writing – review and editing, data curation.

## Conflicts of Interest

The authors declare no conflicts of interest.

## Supporting information

PGX Survey Supplementary file.

## Data Availability

The data that support the findings of this study are available from the corresponding author upon reasonable request.
