# Supplementary material for: Current State of Pharmacogenomic Implementation Into Care for Persons With Cystic Fibrosis
Source: Pediatr Pulmonol. 2025 Aug 8;60(8):e71229. doi: 10.1002/ppul.71229 (PMC12333322; doi:10.1002/ppul.71229)
Supplement: Supplementary file 1 — PGX Survey Supplementary file. [file PPUL-60-0-s001.docx]

**Cystic Fibrosis Pharmacogenomics (PGx) Utilization Survey**

**Page 1:**

**Consent:**

Your participation is voluntary and there will be no negative repercussions if you choose not to participate.  Your completion of the survey will serve as your consent to participate.  Your consent may be withdrawn at any time. All information collected on the survey will be kept confidential by researchers and reported as group data.

Completion of this survey should take you **no more than 10 minutes** to complete. There are no direct benefits to you by participating in this research project.

If you have any questions about this survey and/or research, please contact Cameron McKinzie (cameron.mckinzie@unchealth.unc.edu). The University of North Carolina at Chapel Hill’s IRB designated this research to be exempt from further IRB review according to the federal guidance on human subjects research.

**Background:**

The Cystic Fibrosis Pharmacy/Clinical Pharmacology Alliance for Research and Collaboration (CF-PHARM) is interested to learn more if and how CF centers are utilizing pharmacogenomics in caring for people with CF (pwCF).

1. Are you currently familiar with pharmacogenomics and its use in clinical practice?
   1. No, I don’t know what pharmacogenomics is.
   2. Yes, I know what pharmacogenomics is, but am not familiar with its clinical application.
   3. Yes, I know what pharmacogenomics is and its use in clinical practice, but do not currently use it in my own practice.
   4. Yes, I know what pharmacogenomics is and its use in clinical practice, and have experience with using it in my own practice.

---------------------------------------

**Page 2:**

**For this survey, pharmacogenomics is defined as:**

The study of how an individual person’s genes impact their response to medications. This could include both efficacy and/or toxicity. Pharmacogenomic (PGx) testing can help to predict which patients will experience expected efficacy with a standard dose and/or which patients are at risk for inefficacy or toxicity with standard doses.

1. Other than CFTR genotyping, is your CF center obtaining PGx testing for any drug-gene pairs?
   1. Yes (branch to #3)
   2. No (branch to #5)
2. In your clinical practice, do you obtain PGx testing proactively or reactively? (Proceed to #4)
   1. *Proactive*: PGx testing results available prior to initiation of new medication(s) that may be impacted
   2. *Reactive*: PGx testing results obtained following either lack of or reduced efficacy or experiencing adverse effects related to impacted medication(s)
   3. Mixture of a and b
3. What current barriers, if any, do you perceive that limit your ability to use PGx testing in your clinical practice? (Please select ALL that apply) (proceed to Implementation section)
   1. Cost of testing
   2. Logistical challenges with obtaining PGx testing (ordering test, difficulty using vendors outside of institution, etc.)
   3. Challenges with interpreting and implementing results
   4. Lack of guidance specific to care of people with CF
   5. Other (please specify)
4. Based on this definition and its potential applications, would you consider proactive or reactive PGx testing to potentially be valuable in your clinical practice?
   1. *Proactive*: PGx testing results available prior to initiation of new medication(s) that may be impacted (proceed to question #6)
   2. *Reactive*: PGx testing results obtained following either lack of or reduced efficacy or experiencing adverse effects related to impacted medication(s) (proceed to question #6)
   3. Both a and b (proceed to question #6)
   4. Would not be valuable in my practice (proceed to question #7)
   5. Other (please specify)
5. What barriers, if any, do you perceive that would limit your ability to use PGx testing in your clinical practice?
   1. Cost of testing
   2. Logistical challenges with obtaining PGx testing (ordering test, difficulty using vendors outside of institution, etc.)
   3. Challenges with interpreting and implementing results
   4. Lack of guidance specific to care of people with CF
   5. Other (please specify)
6. Why do you think PGx testing would not be valuable and/or beneficial to your current practice? (Please select ALL that apply)
   1. Cost of testing
   2. Logistical challenges with obtaining PGx testing (ordering test, difficulty using vendors outside of institution, etc.)
   3. Challenges with interpreting and implementing results
   4. Lack of guidance specific to care of people with CF
   5. Other (please specify)

---------------------------------------

**Page 3:**

**For this survey, implementation is defined as:**

PGx testing that is completed and yields results for clinical use (e.g., entered into the health record) which may include infrastructure in place to support test ordering, test interpretation, applying results to drug prescribing, and/or returning results to patients.

1. Is PGx-guided implementation for at least one drug-gene pair implemented into patient care at your institution, or is your institution currently in the planning stage for implementation?
   1. Currently implemented into patient care
   2. In the planning stage to implement into patient care
   3. Not implemented into patient care (i.e., research protocol only)
2. Which of the following patient populations receive (or will receive if in the planning stage) PGx-guided implementation for at least one drug-gene pair?
   1. Adult patients
   2. Pediatric patients
   3. Both
3. How long has infrastructure for PGx testing been available at your institution?
   1. ≤6 months
   2. >6 months-1 year
   3. >1 year-5 years
   4. >5 years
   5. I do not know
   6. Not currently implemented
4. Where is genotyping performed at your institution (or will be performed if in the planning stage)? (Please select ALL that apply)
   1. ‘In-house’ by a clinical laboratory at the institution
   2. Another healthcare institution’s clinical laboratory
   3. A commercial laboratory
   4. A research lab
   5. I do not know
   6. Other (please specify)
5. Which of the following genes are tested **and** used to guide therapy in your CF population (or anticipated to be tested if in the planning stage)? (Please select ALL that apply)
   1. CYP2D6
   2. CYP2C9
   3. CYP2C19
   4. CYP3A5
   5. CYP3A4
   6. UGT1A1
   7. I do not know
   8. Other (please specify)
6. What resources or references do you utilize to aid in interpretation of PGx results? (Please select ALL that apply)
   1. Clinical Pharmacogenetics Implementation Consortium guidelines
   2. PharmGKB
   3. PubMed (primary literature)
   4. Tertiary drug information references
   5. Commercial laboratory-provided guidance
   6. Other (please specify)
7. What clinical decision support or expertise is available at your center for assisting clinicians with interpreting and applying PGx test results to individual patient medication prescribing? (Select ALL that apply)
   1. Formal PGx consult service
   2. PGx clinical pharmacist specialist
   3. Automated EMR-based clinical decision support
   4. Other (please specify)

**Page 4:**

**Demographics:**

- - - 1. **Which of the following BEST describes your role within the CF Center?**

If CF Pharmacist (Pediatric), CF Pharmacist (Adult), CF Pharmacist (Pediatric and Adult) selected, then go to question 2

If one of the following selected then additional question pops up:

CF Center Director (Pediatric), CF Center Director (Adult), CF Center Director (Pediatric and Adult), Physician (Pediatric), Physician (Adult), Physician (Pediatric and Adult), Nurse Practitioner (Pediatric), Nurse Practitioner (Adult), Nurse Practitioner (Pediatric and Adult), Physician Assistant (Pediatric), Physician Assistant (Adult), Physician Assistant (Pediatric and Adult), Other (please specify)

If other, please describe your role within the CF center:

Additional question: Does your center have a pharmacist? Yes/No

1. **Which of the following best describes your CF center?** Affiliate Center, Primary Center, Outreach Center
2. **What CF center do you practice at?**
3. **What CF population are you primarily responsible for?** Peds, Adult, Peds & Adult
4. **How many ADULT patients are cared for within your CF Center? :** <100, 101-150, 151-200, 201-250, 251-300, >300
5. **How many PEDIATRIC patients are cared for within your CF Center?** <100, 101-150, 151-200, 201-250, 251-300, >300
